# Investigation and analysis of maker education curriculum from the perspective of artificial intelligence

**DOI:** 10.1038/s41598-024-52302-1

**Published:** 2024-01-23

**Authors:** Qizhong Ou, Xinglin Chen

**Affiliations:** School of Computer and Information Engineering, Nanning Normal University, Nanning, 530100 Guangxi China

**Keywords:** Computer science, Information technology

## Abstract

As an education model that focuses on the cultivation of students’ creativity and practical ability, the integration of maker education and the development of artificial intelligence is of great significance. In order to analyze and study the application effect of artificial intelligence in maker education, based on this, this study adopts the method of questionnaire and case analysis to investigate and analyze students from three dimensions: learning experience, learning attitude and learning emotion, learning effect and transfer. The survey results show that, on the whole, the integration of artificial intelligence technology into maker education can significantly improve students’ learning feelings and attitudes, and enhance the enthusiasm of learning emotions. The research also finds that it has a positive impact on students’ learning effect and transfer ability. Learning experience, learning attitude and learning emotion, learning effect and learning transfer are related to gender, teaching time, teaching subjects or majors. Therefore, the article will suggest creating a maker cultural atmosphere, creating a maker resource space, and improving the maker education system. This provides a valuable reference for further promoting the development of maker education courses in the field of artificial intelligence.

## Introduction

### Research background

With the rapid development of science and technology and social changes, cultivating creativity and practical ability has become an important goal of contemporary education. As an educational model that emphasizes students’ active participation, practical exploration and teamwork, maker education has been widely concerned and applied. At the same time, artificial intelligence, as a cutting-edge technology, has shown great potential in many fields and has gradually penetrated into the field of education.

The combination of maker education and artificial intelligence has had a huge impact on the education field, and is of great significance for the integration of digital education resources and the balanced development of education. Artificial intelligence technology can provide powerful tool support for maker education, such as intelligent assisted learning systems based on machine learning and natural language processing, programmable robots, etc. These technologies can stimulate students’ creativity and exploration desire, improve their learning effect and motivation.

However, there is currently relatively little application of AI in maker education. Therefore, this study will use the method of questionnaire to deeply analyze the application effect of artificial intelligence in maker education, and evaluate it from multiple dimensions such as learning experience, learning attitude and learning emotion, learning effect and transfer. The research results will provide an important reference for maker education practitioners and education decision-makers to better promote the application of artificial intelligence in maker education and promote the cultivation of students’ creativity and practical ability.

### Research methods

This paper mainly adopts three research methods: literature research, investigation research and case study. First of all, this paper makes an in-depth analysis of the relevant research literature on artificial intelligence and maker education, which provides a solid theoretical support for this study. Secondly, this paper collects the data of maker education curriculum research from the perspective of artificial intelligence, combines the practical application effect, and carries out research, which provides data support for this paper.

## Literature review

### Research status of maker education

The word “Maker” comes from the English dictionary “maker”. It refers to a group of people who turn ideas into reality through the Internet, 3D printing and other technologies^1^. The earliest examples of maker education can be traced back to the late last century’s innovative project FabLab(FabricLaboratory) at MIT’s Center for Research in Bits and Atoms. The main focus of the innovation project is: personal creativity, personal design and personal creation^2^. The rise of maker education abroad, in large part because of President Obama’s speech at the Education Reform Conference in November 2009, the connection between the two is very close and direct, he called on every student to be a creator, not just a consumer^3^. In order to further stimulate the creative talent of every student and enhance their practical ability and innovation awareness and ability, in 2012, the United States launched the “Maker Education Program” (MEI)^4^, which aims to make it possible for every young person to become a maker. The U.S. government held a “Maker Carnival” at the White House in 2014. President Obama called on “people across the country to engage in activities that encourage innovation and encourage social creativity”^5^. to make maker education deeper and more extensive. Of course, in addition to the maker education program, the U.S. Department of Education has also launched MakeOver, a project aimed at creating more makerspaces in K-12 schools to incorporate the ideas and hands-on experience of makers into their education^6^. Of course, in addition to the United States, other Western countries, led by the United Kingdom, Germany, and Japan, are also at the forefront of the development of maker education.

Compared with developed countries, China’s research in maker education is relatively lagging behind, but in 2015, Premier Li Keqiang mentioned the concept of “maker” for the first time in the government work report, and then repeatedly in the relevant documents issued by the Ministry of Education: repeatedly emphasizing the need to cultivate innovative and composite talents, strengthen the cultivation of innovation awareness, innovation ability, and practical ability. It has promoted the development of academic research on maker education in China. For example, the Outline of the National Medium—and Long-Term Education Reform and Development Plan (2010–2020), the Decision of the CPC Central Committee and The State Council on Deepening Education Reform and Comprehensively Promoting Quality Education, etc.^7^.

The establishment of the first makerspace in Shanghai in 2010 was the unveiling of the maker movement in China. Then, in 2013, Mr. Wu Junjie published a paper “Maker Education: Opening Up a New Road for Education”, which for the first time presented the term “maker education” in the view of domestic academics and media^8^. For example, Professor Yang Xianmin explained maker education from two perspectives: the first is “maker education”, that is, through the establishment of maker courses, the establishment of a complete maker space, and the provision of professional teacher development, aimed at cultivating creative talents. Another explanation is “maker education”, which combines the ideas of makers with various courses to change education with the ideas and methods of makers^9^. Zhu Zhiting and other researchers interpret maker education from two dimensions, broad and narrow, broad: maker education is a form of education oriented to cultivate the spirit of makers in the public. In the narrow sense, maker education is an educational model that focuses on cultivating learners, especially young learners’ maker literacy. In the narrow sense, maker education includes formal learning and informal learning^10^.

### Research status of maker education teaching model

At present, Project-Based Learning (PBL) is the main teaching method of maker education^11^. Maker education adopts Learning By Design (LBD) as the main learning method^12,13^. Based on project-based teaching method and design-based learning theory, many researchers have built a maker education teaching model with Chinese characteristics. For example, Chen Gang et al. believe that maker courses belong to the “mountaineering” course with “theme-exploration-expression” as the basic course organization model, which should be designed on the basis of “experience unit”, and the performance evaluation of maker courses should be based on “application layer”. Based on the application of micro-classroom in robot teaching, Professor Wang Tongju built a teaching model of “micro-classroom guidance” based on the application of micro-class in robot teaching^14^. Yang Xiaotong and other researchers put forward the online and offline maker space as the supporting environment to support the primary and secondary school maker teaching model (CMT model for short), and evaluated the effect of this model from two aspects: maker works and students’ own innovation ability^15^. By analyzing the maker education supported by the network “teaching space”, researcher Yang Bin summarized four strategies to carry out maker education in primary and secondary schools, including: phased implementation of maker education strategy, project “group” design strategy, maker works tour strategy, and application of “wechat QR code” to seek creative assistance strategy^16^; Referring to STEM interdisciplinary project design model, Professor Yang Wei et al. proposed the overall framework model of maker education based on subject curriculum integration. The model is based on teaching analysis, centered on the project, and designed from the aspects of teaching activities, teaching resources, teaching structure, and teaching evaluation^17^.

To sum up, at present, the educational community has rich research results on maker education, but the research on the teaching mode of maker education is also different, but more are project-based teaching mode, and relatively few use artificial intelligence technology to integrate into the teaching mode of maker education. Therefore, the research on this topic is more meaningful. Based on this, this study will discuss the investigation and research of maker education curriculum from the perspective of artificial intelligence.

### Ethical approval

This study was conducted in accordance with the Helsinki Declaration and approved by the Human Research Ethics Committee of Nanning Normal University (protocol code/approval number: NNUHR-2022-03). All individual participants in the study received informed consent forms.

## Research platform and data collection

### Superstar platform

Superstar Fanya platform is an online learning platform launched by Beijing Superstar Group, which has both PC and mobile terminals. The PC side is “Superstar Erya”, the mobile side is “learning pass”, and it is a learning platform for mobile terminals such as smart phones and tablets. The two data is interlinked, teachers and students can log in through the web page, but also download the mobile end of the super star learning pass software to use the platform functions. With the promotion of online learning in recent years, the number of use of the Superstar platform has increased year by year. Teachers can build courses, upload course resources, and collect relevant learning data on the PC “Superstar Erya”, while learners can study, download relevant resources, complete discussions, assignments, and examinations on “Superstar Erya” or “Learning Pass”. It can provide more convenient learning experience for learners, break the limitation of time and space, and learn more freely and quickly. At the same time, the platform also has a complete data analysis function, which can show students’ course learning, homework and exams in the form of data charts. By using this function, teachers can better grasp the learning progress of students. At the same time, the Super Star teaching platform provides teachers and students with a series of services before, during and after class, allowing learners to study anytime and anywhere, and bringing a good learning experience for learners.

### Data collection and sample description

Taking the students who choose the course of “Maker Education in primary and secondary schools” as the research object, this part of students were randomly investigated to collect the information needed for the research. The students participating in this course come from different schools and different majors, and the research object covers a wide range.

In this study, questionnaires were distributed through the Internet. In order to ensure the validity of the questionnaire data, the author conducted a pre-survey at the beginning of the questionnaire to check whether the questionnaire structure is reasonable and whether the semantic expression is clear. According to the analysis of the results of the preliminary investigation, the author modified and adjusted the questionnaire for the formal investigation of this study.

### Questionnaire design

The questionnaire was formed by combining the Blue Book of Maker Education in China and the Evaluation Index System of Maker Education Development in Primary and Secondary Schools published by the Education Bureau of Zhengzhou, China, and was repeatedly revised and reviewed, the final questionnaire is divided into four parts: students’ basic situation, students’ learning feelings about the course, learning emotions and attitudes, learning effects and transfer, with a total of 35 questionnaires. The first part aims to collect the background information of the students, including gender, ethnicity, working age and other basic personal information. The second part is about collecting students’ learning feelings about the course teaching. For example, this course has helped me learn about maker education in primary and secondary schools, it is easy for me to master and learn this course, and I think the course is very easy to use. The third part is about students’ learning emotion and attitude, including 17 measurement factors. The fourth part is about the learning effect and transfer of the students, including six measurement factors such as “integration of maker education and discipline”. The students were asked to use a 5-point Likert scale to evaluate their attitudes and opinions on the learning of maker education courses from the perspective of artificial intelligence (1 = strongly disagree, 2 = disagree, 3 = general, 4 = agree, 1 = strongly disagree, 2 = disagree, 3 = agree, 4 = agree, 2 = disagree). 5 = strongly agree)^18–20^.

### Reliability and validity test and analysis

Statistical analysis of the collected data was performed using the analysis package IBM SPSS. At the beginning of data analysis, it is necessary to verify the validity and reliability of the questionnaire. In this paper, Klonbach α reliability coefficient method and KMO measurement value are used to judge. Statistical analysis of the collected data shows that Klonbach α reliability coefficient is 0.966, KMO measurement result is 0.948, and the null hypothesis of Bartlett sphere test is not valid (*p* = 0.000 < 0.05), indicating that the questionnaire has high reliability and validity.

## Research results and analysis

### Analysis of students’ basic situation

The basic analysis of the data is as follows: During the survey period (March–May 2022), a total of 531 questionnaires were collected. After preliminary sorting, invalid questionnaires and irregular data were eliminated, and 490 valid data were obtained with an effective recovery rate of 92.2%. Table 1 shows a descriptive analysis of the feedback collected. Students from the Guangxi region accounted for a larger proportion, 395 (80.16%), while students from outside the Guangxi region accounted for 95 (19.39%). In terms of gender distribution, there were 60 boys (12.24%) and 430 girls (87.76%). The subjects in this study mainly come from different disciplinary backgrounds. Most of the students are in other majors or teaching subjects, such as art and information technology. Relatively few students are engaged in teaching or learning subjects such as Chinese and mathematics. In addition, the object of this study is mainly college students, accounting for the vast majority, 470 people (96%). Only a few students are primary and secondary school teachers. In terms of teaching time, the absolute advantages are non-employed teachers or college students, followed by 1–5 years and more than 10 years.

**Table 1:** Results of student background information.

Variable	Choice	Frequency	Percentage (%)
Ethnicity	Han	343	70
	Zhuang	123	25.1
	Miao	9	1.84
	Dong	2	0.41
	Yao	8	0.2
	Maonan	2	1.63
	Mulao	2	0.41
	Gelao	1	0.41
	Other Ethnicities	0	0
Household registration	Location Guangxi	395	80.61
	Outside Guangxi	95	19.39
Gender	Female	430	87.76
	Male	60	12.24
Teaching subject or major	Subject	49	10
	Math	62	12
	English	32	6
	Physics	7	1.4
	Chemistry	5	1
	Biology	12	2
	Ideological and Moral Education	15	3
	History	9	1.8
	Geography	6	1.2
	Science	2	0.4
	Art	33	6.7
	Integrated Practice	4	0.8
	Physical Education	21	4.2
	Information Technology	27	5
	Other	206	42
Teaching Experience	1–5 years	10	2
	6–10 years	0	0
	More than 10 years	10	2
	Unemployed student	470	96

### Analysis of learning experience

**Figure 1:**
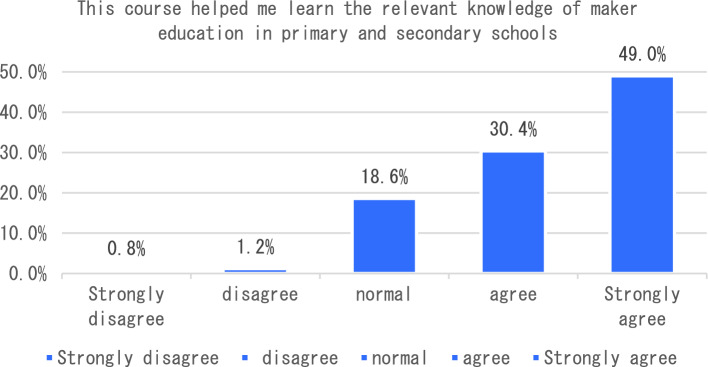
Comparative analysis of “This course helped me learn the relevant knowledge of maker education in primary and secondary schools”.

**Figure 2:**
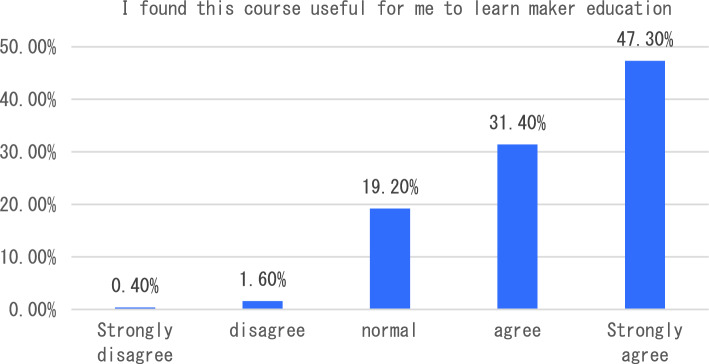
Comparative analysis of “I found this course useful for me to learn maker education”.

**Figure 3:**
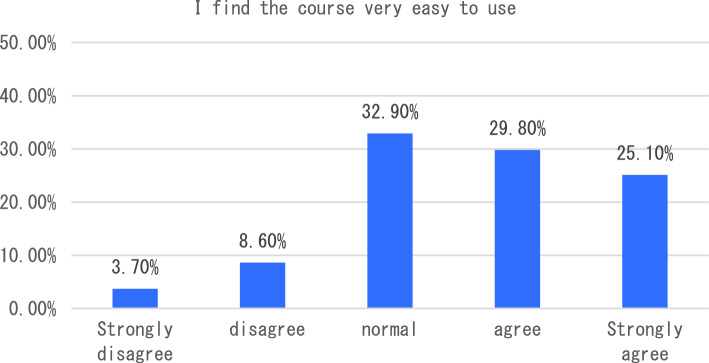
Comparative analysis of “I find the course very easy to use”.

**Figure 4:**
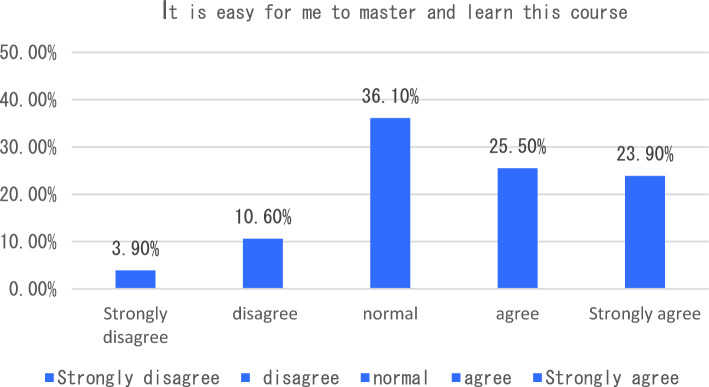
Comparative analysis of “It is easy for me to master and learn this course”.

**Figure 5:**
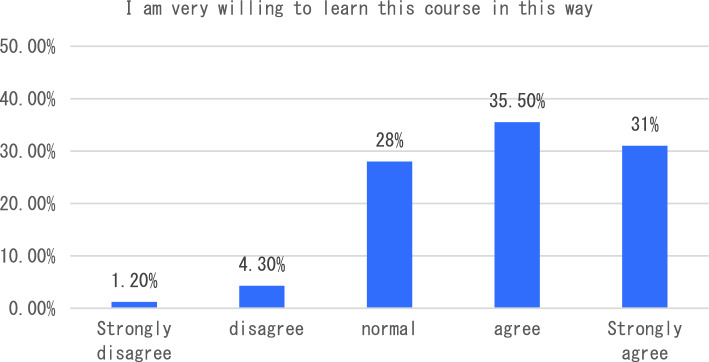
Comparative analysis of “I am very willing to learn this course in this way”.

**Figure 6:**
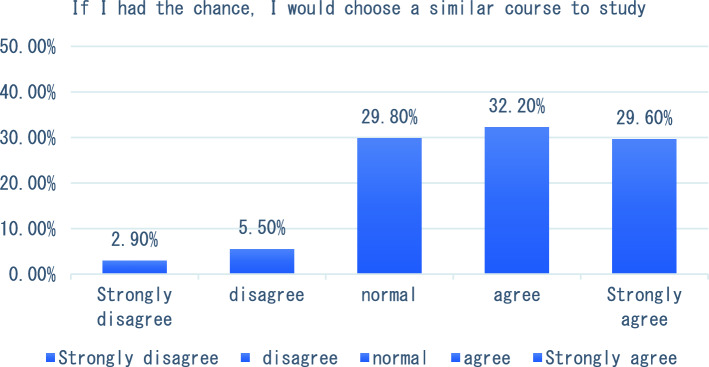
Comparative analysis of “If I had the chance, I would choose a similar course to study”.

With the in-depth application of artificial intelligence in the field of education, more and more researchers have begun to try its maker education courses and carry out corresponding teaching practices. The teaching of maker education from the perspective of artificial intelligence includes six aspects, such as graphic programming workshop and educational robot workshop. In terms of learning experience, we will start with six indicators including how this course helped me learn the relevant knowledge of maker education in primary and secondary schools, and how I found this course very useful for me to learn maker education. The survey results showed that the vast majority of students agreed that the course helped students understand maker education more clearly (see Fig. 1). In terms of the indicator “I found this course very useful for me to learn maker education”, 47.3% of the students think that the course is very useful for them to understand maker education, followed by 31.4% of the students say that the course is useful, and only 2% of the students think that the course is not useful for them to master maker education (see Fig. 2). The survey of students’ feelings about the course found that the majority of students said they agreed or strongly agreed that the course was easy to learn (54.90%). However, a small number of students indicated that they disagreed or strongly disagreed that the course was very easy to use (see Fig. 3). To investigate students’ mastery of the course: Only 14.5% of the students said that the course is relatively difficult for them to understand, and 49.40% of the students said that it is easy for them to master and understand the content of this course (as shown in Fig. 4). This course is different from the previous maker education courses. Artificial intelligence technology is introduced in this course. Help students more easily and comprehensively understand maker education or maker space, so as to cultivate students’ innovative thinking, logical thinking ability, and better improve students’ hands-on ability. As shown in Fig. 5, 67.5% of the students said that they liked the learning method of this course very much, 28% of the students liked it moderately, but they did not reject the teacher’s explanation of relevant theoretical knowledge, and only 5.7% of the students said that they did not like this learning method and were not willing to use this learning method to learn makers. This course is the first attempt to integrate artificial intelligence technology into the teaching of maker education. Different from previous teaching of maker education, this form of teaching integration can provide students with a new learning experience and further deepen their understanding of makers. In order to further explore students’ attitude towards this integration (as shown in Fig. 6), 61.8% of the students said they would choose a similar course if given the chance. Only 7.4% of students said they would not choose a similar course of study. From the data in Figs. 1, 2, 3,4, 5 and 6, the introduction of artificial intelligence technology in maker education teaching not only provides students with a new learning experience, but also stimulates students’ creativity and problem-solving ability. This integrated approach to teaching has had a positive impact on students’ learning. At the same time, it also provides opportunities for them to better understand and grasp maker education.

### Analysis of learning attitude and emotion

In this study, mean M and variance SD are used to characterize the learning attitudes and emotions of students who take the course “Maker Education in Primary and Secondary Schools” towards a specific item. Through the analysis of Table 2, it is found that:

**Table 2:** Descriptive statistical analysis of learning attitude and emotion.

Descriptive statistics		
	Mean	Variance
The learning tasks and content in the course attract my attention	4.00	.806
I do not feel bored while learning in this course	3.89	.845
The learning activities and materials in the course stimulate my curiosity	4.07	.717
I have learned a lot of useful things in this course	4.15	.686
The course content is related to my own learning goals and expectations	3.97	.784
The course supplies some content related to my work or experience	3.89	.846
I did well in this course	3.74	.915
I am satisfied with my performance in the course	3.79	.848
I worked hard enough and learned well in this course	3.89	.833
I enjoy learning this course	3.93	.813
I am satisfied with the knowledge I have learned in this course	3.99	.777
Completing the learning tasks in the course makes me feel very satisfied	4.01	.769
I feel stressed while learning the course	3.65	.916
I often worry whether I can complete the tasks	3.73	.963
I often feel nervous and want to give up during the course learning process	3.24	1.284
This course can supply more educational opportunities for people in remote areas	4.06	.751
This course supplies equal educational opportunities for everyone	4.11	.762

Learning attitude and emotion of maker education courses from the perspective of artificial intelligence: It can be seen from the survey results that students hold a positive attitude towards most of the analysis dimensions of the course, with the average concentration between 3.7 and 4.2, and the variance is relatively small, indicating that students’ answers to each index are relatively consistent. However, there are also some differences in a few indicators, among which the most significant one is that under the indicator “I often feel nervous and want to give up during the course learning”, the variance is large, indicating that students have diversified views on this indicator. On the whole, the introduction of artificial intelligence technology in maker education teaching can not only change and improve students’ learning attitude, but also enhance students’ enthusiasm for learning emotions. At the same time, teachers also need to pay attention to the emotional state of students and provide corresponding help and guidance when necessary to ensure that they can overcome difficulties and continue to learn.

### Learning effect and transfer analysis

**Figure 7:**
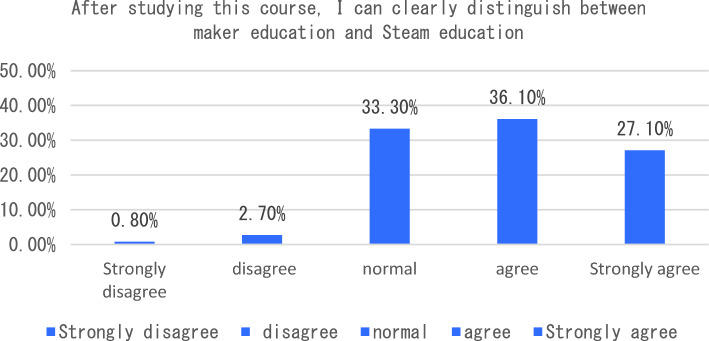
Comparative analysis of “After studying this course, I can clearly distinguish between maker education and Steam education”.

**Figure 8:**
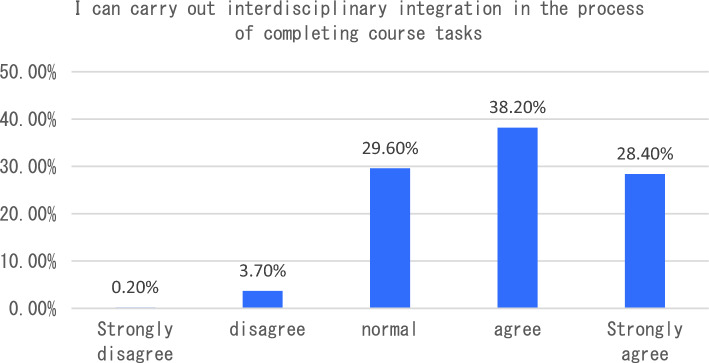
Comparative analysis of “I can carry out interdisciplinary integration in the process of completing course tasks”.

**Figure 9:**
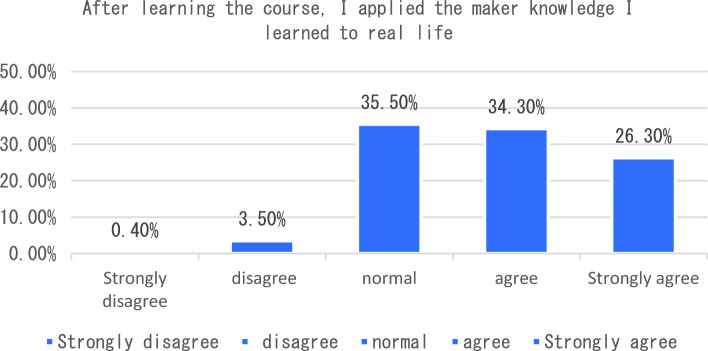
Comparative analysis of “After learning the course, I applied the maker knowledge I learned to real life”.

**Figure 10:**
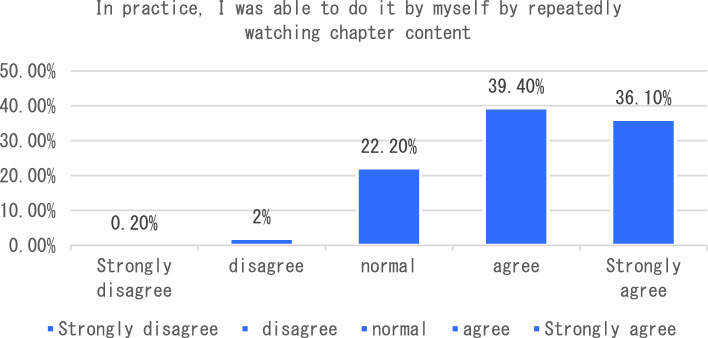
Comparative analysis of “In practice, I was able to do it by myself by repeatedly watching chapter content”.

**Figure 11:**
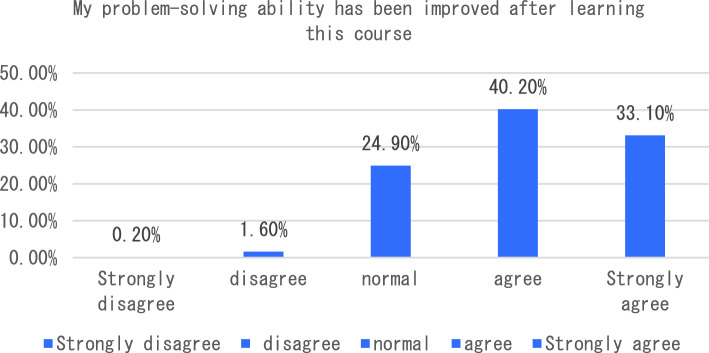
Comparative analysis of “My problem-solving ability has been improved after learning this course”.

**Figure 12:**
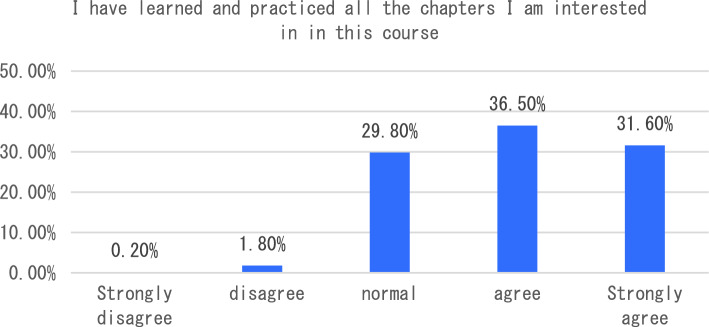
Comparative analysis of “I have learned and practiced all the chapters I am interested in in this course”.

The dimension of learning effect and transfer is that individuals consciously pay attention to their own learning effect. In terms of learning effect and transfer, I will be able to carry out interdisciplinary integration in the process of completing the course tasks. After completing the course, I will apply the maker knowledge I have learned to my other disciplines and analyze six sub-dimensions. The survey shows that the vast majority of students can clearly distinguish between maker education and STEAM education after learning the course, 33% of students said that as before, only 3.5% of students can not clearly distinguish the correlation and distinction between the two (see Fig. 7). In the process of completing the course tasks, I was able to conduct a comparative analysis of interdisciplinary integration, as shown in Fig. 8. From the analysis of the survey results, we can see that 66% of the students chose to be able to carry out interdisciplinary integration, while only a small number of students could not deeply integrate the knowledge of maker education with other disciplines. As an education mode that focuses on the cultivation of students’ creativity and practical ability, maker education emphasizes the cultivation of students’ hands-on practical ability and problem-solving ability. From the data presented in Figs. 9, 10, 11 and 12, it can be seen that most students’ hands-on ability and problem-solving ability have been improved and the knowledge they have learned about making makers has been applied to real life. However, there are still a small number of people who have a small space span for the improvement of creativity and practical ability. Therefore, teachers can provide more appropriate encouragement and guidance in the future courses.

### Difference analysis of learning experience, learning attitude and emotion, learning effect and transfer

In this study, Pearson correlation analysis was used to examine the differences of students’ learning experience, learning attitude and emotion, learning effect and transfer in Maker Education for primary and secondary schools.

#### Difference analysis of learning experience

The results of correlation analysis show that the students’ perception of intelligent learning is related to their gender, teaching age, major or teaching subject.

As shown in Table 3, students with different genders, majors or teaching subjects and teaching years have different learning feelings. It can be seen that: (1) In terms of gender, male and female students have no obvious difference in learning feelings. In particular, “This course helped me learn the relevance of maker education in primary and secondary schools” showed negative correlation. (2) In terms of majors or teaching subjects, students of different majors have little discussion on the learning of maker education; The six indicators of learning experience were all negatively correlated. (3) Compared with the older students, the younger students are more likely to agree that AI technology and the teaching mode of maker education help students understand the knowledge of maker education (*p* = 0.043, r = − 0.027) and help students understand the practicability of the course (*p* = 0.043, r = − 0.002), help students master the course content (*p* = 0.003, r = − 0.0133), think the course is easier to learn (*p* = 0.001, r = − 0.144), more willing to adopt this learning style (*p* = 0.045, r = − 0.091), are more willing to continue studying similar courses (*p* = 0.031, r = − 0.097).

**Table 3:** The difference of learning experience of students with different gender, major or teaching subject and teaching age.

Dimensionality	Subdimension	Gender		Major or teaching subject		Length of teaching	
		*p*	r	*p*	r	*p*	r
Learning experience	This course helped me learn about maker education in primary and secondary schools	0.043	− 0.027	0.002	− .138**	0.162	− 0.063
	I found this course to be especially useful for learning about maker education	0.043	− 0.002	0.064	− 0.084	0.103	− 0.074
	The course is extremely easy to get started with	0.001	− 0.144	0.78	− 0.013	0.015	− .110
	It was easy for me to grasp and learn from this course	0.003	− .0133	0.858	− 0.008	0.019	− .106
	I am very willing to learn this course through this type of learning method	0.045	− .091	0.021	− .104*	0.224	− 0.053
	If given the opportunity, I would choose to learn similar courses again	0.031	− .097	0.019	− 0.679	0.136	− 0.0067

Different numbers of stars have different meanings, with one star indicating *p* < 0.05 and having a significant correlation; These two asterisks indicate *p* < 0.01 and the correlation is very significant.

#### Analysis of differences between learning attitude and emotion

The results of correlation analysis show that the students who choose Maker Education in primary and secondary schools have a certain correlation between their learning attitude and emotion and gender, teaching age, major or teaching subject. The differences in learning attitudes and emotions of students of different genders, majors or teaching subjects and teaching ages are shown in Table 4. It can be seen that: There is no significant difference between male and female students in learning attitude and clarity of learning. However, I do not feel bored when I study this course, and the course provides some content related to my work or experience, showing a negative correlation, which indicates that male and female students have certain differences in the boredom of the course and the feeling of the content related to work or experience. There is no difference in the learning attitude and clarity of maker education in different majors or teaching subjects. The main difference is that the nature of the discipline will affect the difference in students’ attitude and emotion towards learning, and the engineering discipline is more willing to participate in the course learning. It’s also easier to feel satisfied. Different teaching hours have no difference in learning attitude and emotion. The main difference is that students with shorter teaching age pay more attention to learning and understanding the course content.

**Table 4:** Differences in learning attitudes and emotions among students of different genders, majors or teaching subjects and teaching ages.

Subdimension	Gender		Major or teaching subject		Length of teaching	
	*p*	r	*p*	r	*p*	r
The learning tasks and content in the course attract my attention	0.179	− 0.061	0.013	− .112*	0.146	− 0.066
I do not feel bored while learning in this course	0.02	− .105*	0.101	− 0.074	0.043	− .092*
The learning activities and materials in the course stimulate my curiosity	0.876	− 0.007	0.5	− 0.031	0.14	− 0.067
I have learned a lot of useful things in this course	0.174	− 0.062	0.089	− 0.077	0.107	− 0.073
The course content is related to my own learning goals and expectations	0.121	− 0.07	0.127	− 0.069	0.127	− 0.069
The course supplies some content related to my work or experience	0.002	− .141**	0.071	− 0.082	0.052	− 0.088
I did well in this course	0.018	− .107*	0.308	− 0.046	0.031	− .097*
I am satisfied with my performance in the course	0.14	− 0.067	0.224	− 0.055	0.108	− 0.073
I worked hard enough and learned well in this course	0.408	− 0.037	0.389	− 0.039	0.17	− 0.062
I enjoy learning this course	0.274	− 0.05	0.066	− 0.083	0.058	− 0.086
I am satisfied with the knowledge I have learned in this course	0.669	− 0.019	0.209	− 0.057	0.057	− 0.086
Completing the learning tasks in the course makes me feel very satisfied	0.813	− 0.011	0.006	− .125**	0.069	− 0.082
I feel stressed while learning the course	0.327	0.044	0.449	0.034	0.092	0.076
I often worry whether I can complete the tasks	0.346	0.043	0.854	− 0.008	0.174	0.062
I often feel nervous and want to give up during the course learning process	0.768	− 0.013	0.307	0.046	0.159	0.064
This course can supply more educational opportunities for people in remote areas	0.848	− 0.009	0.05	− 0.088	0.381	− 0.04
This course supplies equal educational opportunities for everyone	0.709	0.017	0.076	− 0.08	0.171	− 0.062

Different numbers of stars have different meanings, with one star indicating *p* < 0.05 and having a significant correlation; These two asterisks indicate *p* < 0.01 and the correlation is very significant.

#### Difference analysis of learning effect and transfer

The results of correlation analysis show that the students who choose Maker Education in primary and secondary schools have a certain correlation with their gender, teaching age, major or teaching subject. The differences in learning effect and transfer between students of different genders, majors or teaching subjects and teaching age are shown in Table 5. It can be seen that there is no obvious difference between male and female students in learning effect and transfer. The only difference is that male students are more willing to apply the maker knowledge they have learned to their real life. There is basically no difference between the learning effect and transfer of maker education in different majors or teaching subjects. It is worth noting that there is a negative correlation between different majors in completing maker projects independently. This means that there are certain differences between students of different majors in completing maker projects independently. Different teaching time has no difference in learning effect and transfer. The main difference is that students with shorter teaching age are better at applying theory to real life.

**Table 5:** The differences of learning effect and transfer among students of different gender, major or teaching subject and teaching age.

Subdimension	Gender		Major or teaching subject		Length of teaching	
	*p*	r	*p*	r	*p*	r
After completing this course, I can clearly distinguish between maker education, STEAM education, and robotics education	0.104	− 0.074	0.988	0.001	0.381	− 0.04
During the course tasks, I can integrate multiple disciplines to complete my works to the best of my ability	0.172	− 0.062	0.592	− 0.024	0.381	− 0.057
After learning the knowledge of maker education, I have applied it to my teaching practice with satisfactory results	0.01	− 0.011**	0.38	− 0.04	0.049	− 0.89
In the practical process, I can independently solve knowledge difficulties by repeatedly watching chapter content	0.803	− .0011	0.025	− .102	0.304	− 0.047
My critical thinking skills have improved after studying this course	0.445	− .034	0.058	− 0.086	0.433	− 0.035
I have learned and effectively applied all chapters that interest me in this course	0.289	− .048	0.643	− 0.021	0.185	− 0.06

Different numbers of stars have different meanings, with one star indicating *p* < 0.05 and having a significant correlation; These two asterisks indicate *p* < 0.01 and the correlation is very significant.

## Teaching practice case analysis of maker education curriculum from the perspective of artificial intelligence

Because the maker course from the perspective of artificial intelligence integrates multi-disciplinary content, and the production process is more complex than the average project, the entire project may take more than 6 h to complete in the actual teaching process. In this process, the flipped classroom teaching method will be integrated, and with the help of “Internet + resources”, the overall arrangement of the learning process will be more conducive to cultivating students’ maker skills and literacy. Specifically, the following steps can be taken:

### Excitement before class: complete the self-learning task list

Based on the learning conditions of maker education, the method of flipped classroom is introduced to advance theoretical learning. Before class, students are allowed to watch the teaching micro-video of Mid-Autumn Festival and National Day mooncake, understand and master the production principle of Mid-Autumn Festival and National Day mooncake and the details to pay attention to, and know why and why. At the same time, key steps or puzzles are recorded and preliminary design ideas are drawn up. Transfer to the classroom for practice and solution. Classroom research projects are mainly based on group cooperation and exploration, supplemented by teachers’ instructions. Teachers play the role of guides and motivators, encourage students to learn independently and help each other, and build team PK mechanism and seal feedback incentive mechanism.

### Classroom test: interactive Q&A

The makerspace is prepared with sufficient equipment and equipment, so that students can verify the preliminary design ideas drawn up before class, and teachers can test the effectiveness of students’ independent learning on site. The difficulty of detection is comparable to the self-learning task list and teaching video, and the main purpose is to help students experience a sense of achievement in learning, so as to prepare psychologically for the next advanced learning challenge, rather than the regular checking of omissions and shortcomings. Such as classroom test content can be: according to the Mid-Autumn Festival and National Day moon cake instructions, Mid-Autumn Festival and National Day moon cake production ideas, production steps. Interactive content can raise its own questions based on the available material, environment or learning foundation.

### Requirement learning: make works in groups and layers

According to the specific situation of the classroom test, the teacher will clear the obstacles of the basic knowledge that the students must master. For example, when designing Mid-Autumn Festival and National Day mooncakes, teachers can demonstrate the basic operation methods of 3D modeling, but they need not be limited to 3D printing production, allowing students to use other materials for production, and telling them that the key point is to design different elements of Mid-Autumn Festival and National Day mooncakes. For example: asymmetrical graphics and cartoon graphics in the production of the difference, the rest of the part can be divided by different learning levels of students freely, collaborative exploration, independent choice to redesign, free creation or follow the example operation. Therefore, from imitation innovation, innovation and then to the original, gradually improve the students’ innovation ability. In this way, each student can obtain a suitable learning method and succeed in the classroom.

### Feedback improvement: debugging design of the work

No work is perfect from the beginning, but it is very likely to face failure. Students are often excited at the completion of an initial design, but failure threatens to undermine their self-confidence. Therefore, it is necessary for teachers to pass on the craftsman spirit of searching for reasons, seeking breakthroughs, striving for perfection and continuous improvement to students through continuous feedback. For example, the Mid-Autumn Festival and National Day mooncake design has undergone 9 steps of design modification, and it is possible to continue to adjust.

### Teaching evaluation: quantitative evaluation and qualitative evaluation

The combination of formative and summative evaluation can not only make students pay more attention to experience the whole operation process, but also ensure the fairness of the evaluation process.

## Discussion and suggestions

The research results show that the introduction of artificial intelligence technology into maker education teaching can significantly improve students’ learning feelings and attitudes, improve their enthusiasm for learning emotions, and improve their learning effect and learning transfer ability, so as to promote the training of students’ hands-on ability and problem-solving ability. In addition, there is a certain correlation to their gender, teaching age and teaching subjects. For example, the learning experience of students with longer teaching age is slightly cold, and the difference between male and female students in learning experience is not obvious. Based on the above conclusions, this study makes the following recommendations for future maker education programs:

### Create a maker culture atmosphere

Educational environment and atmosphere are also an important part of educational resources. A good maker atmosphere can promote students to recognize and love “maker” thinking, and fully mobilize students’ creativity and initiative. The application of artificial intelligence technology in the field of maker education can provide more practical opportunities for students, and is of great significance to promote the integration of students’ teaching and help students form a true creative spirit and consciousness. At present, many students who receive maker education are in the critical period of knowledge exploration. When they receive maker education, they often face the contradiction between knowledge reserve and learning enthusiasm, which makes these students encounter many difficulties in the process of learning related algorithms and models.

The application of artificial intelligence technology provides a good practice platform for these students, so that students have more opportunities to “create”, “innovate” and “try”, which is of great help to mobilize students’ enthusiasm for learning and cultivate students’ logical thinking, critical thinking, innovative thinking, hands-on ability, brain ability and creative ability. Therefore, maker education should make full use of teaching resources and teaching platforms provided by artificial intelligence, such as Superstar Erya, simulation lab, 3D lab, Arduino platform, etc., to provide students with personalized learning, design, production, and innovation space.

### Create a maker resource space

The core goal of maker education is to cultivate students’ maker spirit and innovative practice ability. To ensure that students have the basic resources that can be created, such as learning resources and practice resources, is the basis of completing the goal of talent training.

Building resource space requires the cooperation of the government, social enterprises, schools and other aspects. The government needs to provide policy, human and material support and guidance for education. For example, to provide some financial support for the development of school maker education, to help link school maker resources with school maker resources and so on. Schools need to deeply understand students’ own characteristics and development interests, and develop school-based resources suitable for maker education. For example, schools can make full use of resource platforms such as technology parks, experimental and training centers, professional libraries, and research-oriented universities within the campus to create school-based or regional maker resource Spaces. Social enterprises should strengthen cooperation with schools and students, and provide channels and other assistance for the transformation of ideas into products on the basis of protecting the interests of enterprises.

### Improve the maker education system

In order to make creative science education can truly train creative talents who can transform creative ideas into products, in addition to creating external conditions such as cultural atmosphere and resource space, it is finally necessary to return to education itself. To improve the maker education system, teachers are first required to have a deep understanding of the core concept of makers, and set the content and form of the course according to the actual needs of students, focusing on the cross-cutting, advanced and integrated curriculum characteristics of maker education. Secondly, teachers should pay attention to the balance of “teaching and doing”. In the aspect of “teaching”, teachers should abandon the traditional teaching mode of infusing or lecturing, and play the role of “enlightener” and “guide”. In the aspect of “learning”, we should pay attention to the mobilization of students’ enthusiasm and initiative, and introduce such classroom learning forms as inquiry, discussion and collaboration. In terms of “doing”, it is necessary to create rich and diversified forms of practice, such as online + offline, in-class + extracurricular forms. It is better to optimize the existing course evaluation methods. In the context of artificial intelligence, curriculum evaluation should meet the needs of students’ personalized and diversified development. The process, dynamic, personalized and data-oriented evaluation methods such as project evaluation, process evaluation and parameter evaluation can be appropriately adopted to make the course feedback obtained by students more objective and comprehensive, reflecting the characteristics of scientific, comprehensive and sustainable maker education.

## Data Availability

All data supporting the findings of the current study are available from the corresponding author upon reasonable request.
